# Evidence of Intercontinental Spread and Uncommon Variants of Low-Pathogenicity Avian Influenza Viruses in Ducks Overwintering in Guatemala

**DOI:** 10.1128/mSphere.00362-16

**Published:** 2017-04-05

**Authors:** Ana S. Gonzalez-Reiche, Martha I. Nelson, Mathew Angel, Maria L. Müller, Lucia Ortiz, Jayeeta Dutta, Harm van Bakel, Celia Cordon-Rosales, Daniel R. Perez

**Affiliations:** a Centro de Estudios en Salud, Universidad del Valle de Guatemala, Guatemala City, Guatemala; b Department of Population Health, Poultry Diagnostic and Research Center, College of Veterinary Medicine, University of Georgia, Athens, Georgia, USA; c Fogarty International Center, National Institutes of Health, Bethesda, Maryland, USA; d Department of Veterinary Medicine, University of Maryland—College Park, College Park, Maryland, USA; e Department of Genetics and Genomic Sciences, Icahn School of Medicine at Mount Sinai, New York, New York, USA; Emory University School of Medicine

**Keywords:** Central America, avian viruses, host range, viral evolution

## Abstract

Over a hundred species of aquatic birds overwinter in Central America’s wetlands, providing opportunities for the transmission of influenza A viruses (IAVs). To date, limited IAV surveillance in Central America hinders our understanding of the evolution and ecology of IAVs in migratory hosts within the Western Hemisphere. To address this gap, we sequenced the genomes of 68 virus isolates obtained from ducks overwintering along Guatemala’s Pacific Coast during 2010 to 2013. High genetic diversity was observed, including 9 hemagglutinin (HA) subtypes, 7 neuraminidase (NA) subtypes, and multiple avian IAV lineages that have been detected at low levels (<1%) in North America. An unusually large number of viruses with the rare H14 subtype were identified (*n* = 14) over two consecutive seasons, the highest number of H14 viruses ever reported in a single location, providing evidence for a possible H14 source population located outside routinely sampled regions of North America. Viruses from Guatemala were positioned within minor clades divergent from the main North American lineage on phylogenies inferred for the H3, H4, N2, N8, PA, NP, and NS segments. A time-scaled phylogeny indicates that a Eurasian virus PA segment introduced into the Americas in the early 2000s disseminated to Guatemala during ~2007.1 to 2010.4 (95% highest posterior density [HPD]). Overall, the diversity detected in Guatemala in overwintering ducks highlights the potential role of Central America in the evolution of diverse IAV lineages in the Americas, including divergent variants rarely detected in the United States, and the importance of increasing IAV surveillance throughout Central America.

**IMPORTANCE** Recent outbreaks of highly pathogenic H7N3, H5Nx, and H7N8 avian influenza viruses in North America were introduced by migratory birds, underscoring the importance of understanding how wild birds contribute to the dissemination and evolution of IAVs in nature. At least four of the main IAV duck host species in North America migrate through or overwinter within a narrow strip of Central America, providing opportunities for diverse IAV lineages to mix and exchange gene segments. By obtaining whole-genome sequences of 68 IAV isolates collected from migratory waterfowl in Guatemala (2010 to 2013), the largest data set available from Central America to date, we detected extensive viral diversity, including gene variants rarely found in North America and gene segments of Eurasian origin. Our findings highlight the need for increased IAV surveillance across the geographical span of bird migration flyways, including Neotropical regions that have been vastly undersampled to date.

## Abstract (precis)

Recent outbreaks of highly pathogenic H7N3, H5Nx, and H7N8 avian influenza viruses in North America were introduced by migratory birds, underscoring the importance of understanding how wild birds contribute to the dissemination and evolution of IAVs in nature. At least four of the main IAV duck host species in North America migrate through or overwinter within a narrow strip of Central America, providing opportunities for diverse IAV lineages to mix and exchange gene segments. By obtaining whole-genome sequences of 68 IAV isolates collected from migratory waterfowl in Guatemala (2010 to 2013), the largest data set available from Central America to date, we detected extensive viral diversity, including gene variants rarely found in North America and gene segments of Eurasian origin. Our findings highlight the need for increased IAV surveillance across the geographical span of bird migration flyways, including Neotropical regions that have been vastly undersampled to date.

## INTRODUCTION

Influenza A viruses (IAVs) have evolved into genetically distinct North American and Eurasian lineages, owing to the natural geographical separation of host species. More recently, expanded surveillance in wild aquatic birds (IAVs’ main natural reservoir) of previously undersampled locations has revealed additional lineages, in South America, Oceania, and Antarctica (1–4). These newly identified lineages highlight significant gaps in our understanding of the ecology and geographic spread of IAVs and the need for additional virus surveillance in understudied regions (5–10). Additional virus surveillance is also important as low-pathogenicity avian influenza viruses (LPAIVs) carried by aquatic birds have been continually identified as the precursors of highly pathogenic avian influenza (HPAI) outbreaks in poultry, including most recently the 2012 outbreak of H7N3 in Mexico and the H5Nx and H7N8 outbreaks in North America during 2014 to 2016 (11–13).

In Guatemala, it is estimated that over 140 aquatic bird species, representing 24 families, are found, 80% of which are migratory or transient visitors (14). More than 123 species can be found in the wetlands along the Pacific Coast. Here, 15 duck species (family Anatidae) have been reported, 10 of which are migratory and include several of the main North American reservoirs of IAV, such as northern shovelers (*Anas clypeata*), northern pintails (*Anas acuta*), blue-winged teals (*Anas discors*), and American wigeons (*Anas americana*) (15). Among these species, blue-winged teals are particularly abundant on the Pacific coast of Guatemala, with counts of >10,000 individuals, whereas the other species are less frequently reported (counts of <250) (14). Other IAV hosts such as green-winged teals (*Anas crecca*), cinnamon teals (*Anas cyanoptera*), and mallards (*Anas platyrhynchos*) also have been reported in Guatemala but are considered migratory vagrants because of their rare appearances during the overwintering period in Central America (14). Due to its particular shape of the landmass, Central America acts as a corridor where multiple bird populations congregate during migration (16). At other latitudes, such conditions are associated with increased virus diversity (17, 18), as interspecies transmission and reassortment reach a peak (19). As it is an overwintering site for several important IAV hosts, virus transmission and reassortment events occurring in Guatemala have the potential to contribute to the virus diversity and extensive gene flow observed in North America (20–22).

Surveillance for low-pathogenicity avian influenza viruses (LPAIVs) has been conducted in wild birds in Guatemala since 2008. LPAIVs from the North American lineage were first detected in overwintering blue-winged teals in Guatemala during 2009 and 2010 (23). During 2010 and 2013, LPAIVs were detected in samples from 11 hunted bird species of the families Anatidae, Columbidae, Rallidae, Scolopacidae, and Threskiornithidae (24). However, the majority of samples were collected from blue-winged teals, from which LPAIVs were detected at various frequencies throughout and between migration seasons. Sixty-eight viruses from 9 hemagglutinin (HA) and 7 neuraminidase (NA) subtypes were isolated from dabbling ducks, including blue-winged teals (*n* = 61), northern shovelers (*n* = 5), a green-winged teal (*n* = 1), and an American wigeon (*n* = 1). To further understand the evolutionary dynamics of LPAIV in Guatemala, we obtained whole-genome sequences from these 68 viruses and performed a phylogenetic analysis. We found evidence of reassortment among viruses sourced from different locations and bird populations, including rarely detected lineages with source populations possibly located in Central America or other undersampled regions.

## RESULTS

### Detection of mixed virus infections.

The full-length genomes of the 68 isolates were obtained by next-generation sequencing (see Materials and Methods). After sequencing, we detected five samples (7%) with mixed infections at the level of the surface glycoprotein genes and a total of 11 mixed infections (16%) when including those with mixed internal gene segments. One of these viruses, an H14 with 2 PA segments, has been previously reported (25). All mixed infections were detected in samples from blue-winged teals (*n* = 10) with the exception of one which was detected in a sample from a green-winged teal.

### Most LPAIVs found in dabbling ducks overwintering in Guatemala are positioned within the North American lineage.

**FIG 1:**
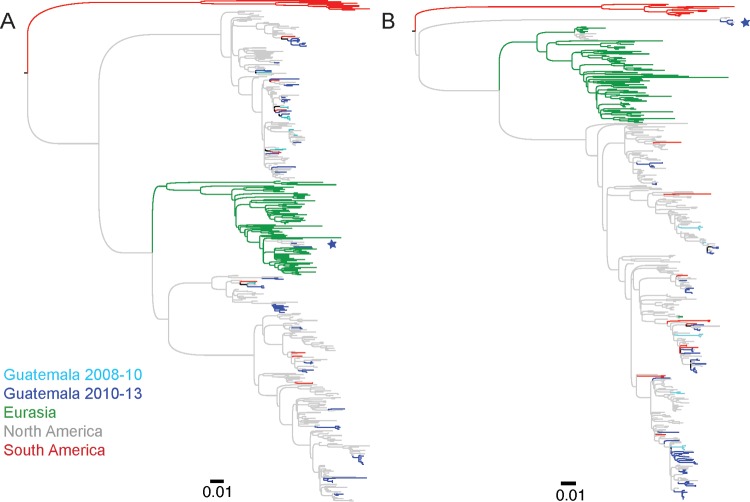
Phylogenetic relationships of PA and NP segments. Phylogenies inferred using maximum likelihood methods for the PA (A) and NP (B) gene segments of LPAIV from Guatemala and other global viruses collected between 2000 and 2013. Viruses are shaded by geographic location. The tree is midpoint rooted, and all branch lengths are drawn to scale. The introduction of Eurasian viruses into North America and Guatemala is indicated with a star on the PA tree. A cluster of divergent viruses from North America and Guatemala that is positioned basal to the diversity of Eurasian and North American viruses is indicated with a star on the NP tree. Detailed ML phylogenies for PA, NP, and additional internal viral protein-encoding gene segments (PB2, PB1, M, and NS) are shown in Fig. S1 to S3.

Phylogenetic analysis by the maximum likelihood (ML) method of the internal gene segments revealed that the Guatemalan viruses, in general, are closely related to other contemporary strains of North American origin (Fig. 1; see also Fig. S1 to S3 in the supplemental material). In agreement with previous observations (23), for all these genes, the viruses from Guatemala are spread among multiple clades of the North American lineage. The viruses are positioned in the phylogenetic trees close to viruses from different locations, including viruses of North American origin that have been recovered in South America and viruses recovered across the United States and Canada (Fig. S1 to S3). The absence of monophyletic groups of Guatemala viruses separated by long branch lengths on the trees provides an indication of the connectivity with avian hosts from locations along different migration flyways.

10.1128/mSphere.00362-16.1 FIG S1  Detailed maximum likelihood phylogenetic inference of the PB2 and PB1 gene segments of LPAIV from Guatemala and other global viruses collected between 2000 and 2013. The tree is midpoint rooted, and branches are drawn to scale. Bootstrap values are shown. The phylogenetic clades (>70% bootstrap clade support) where the viruses from Guatemala are positioned are indicated with Roman numerals. The subclade of the viruses with the most divergent genomes is highlighted in orange. Download FIG S1, PDF file, 0.9 MB. Copyright © 2017 Gonzalez-Reiche et al. 2017 Gonzalez-Reiche et al. This content is distributed under the terms of the Creative Commons Attribution 4.0 International license.

10.1128/mSphere.00362-16.2 FIG S2  Detailed maximum likelihood phylogenetic inference of the PA and NP gene segments of LPAIV from Guatemala and other global viruses collected between 2000 and 2013. The tree is midpoint rooted, and branches are drawn to scale. Bootstrap values are shown. The phylogenetic clades (>70% bootstrap clade support) where the viruses from Guatemala are positioned are indicated with Roman numerals. NA, North American lineage; EA, Eurasian lineage. Color annotations are the same as those for Fig. S1. The subclade of the viruses with the most divergent genomes is highlighted in orange. Download FIG S2, PDF file, 1 MB. Copyright © 2017 Gonzalez-Reiche et al. 2017 Gonzalez-Reiche et al. This content is distributed under the terms of the Creative Commons Attribution 4.0 International license.

10.1128/mSphere.00362-16.3 FIG S3  Detailed maximum likelihood phylogenetic inference of the M and NS gene segments of LPAIV from Guatemala and other global viruses collected between 2000 and 2013. The tree is midpoint rooted, and branches are drawn to scale. Bootstrap values are shown. The phylogenetic clades (>70% bootstrap clade support) where the viruses from Guatemala are positioned are indicated with Roman numerals. For the NS alleles, A and B are indicated in the clade notation. Color annotations are the same as those for Fig. S1. The subclade of the viruses with the most divergent genomes is highlighted in orange. Download FIG S3, PDF file, 0.9 MB. Copyright © 2017 Gonzalez-Reiche et al. 2017 Gonzalez-Reiche et al. This content is distributed under the terms of the Creative Commons Attribution 4.0 International license.

### LPAIVs found in dabbling ducks overwintering in Guatemala during 2010 to 2013 represent 52 genome constellations.

**FIG 2:**
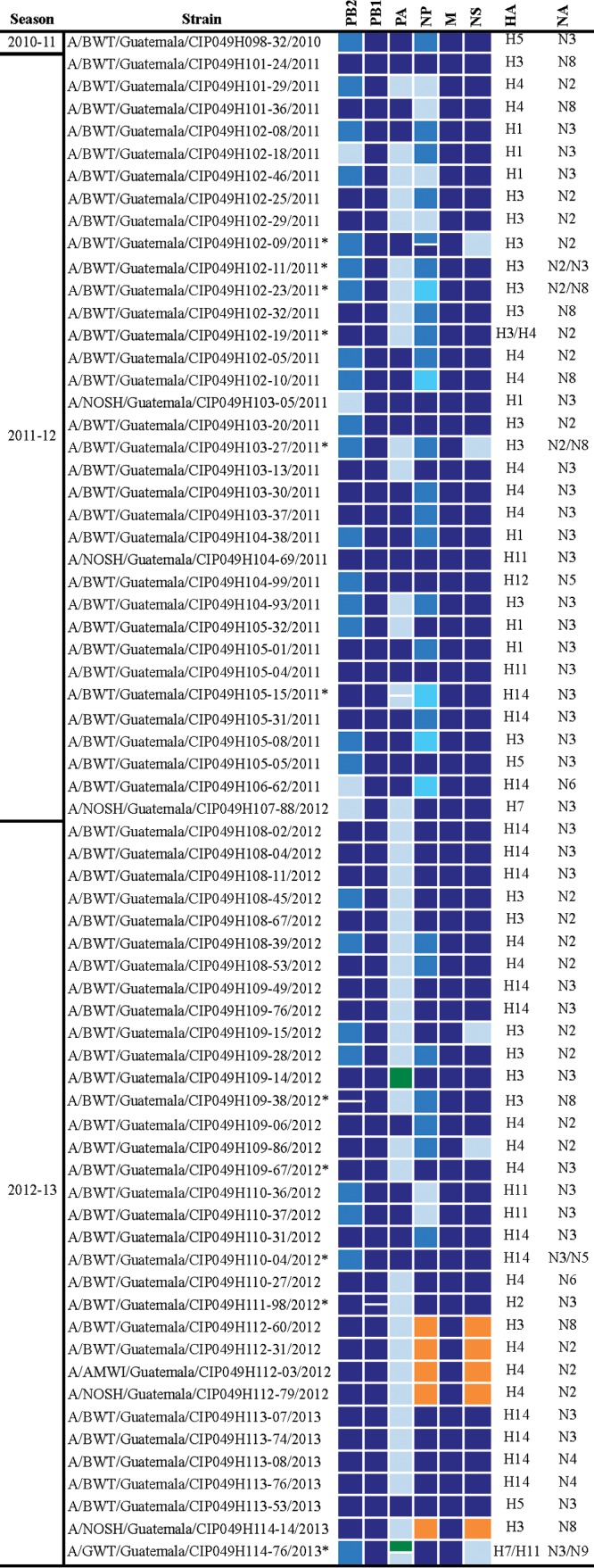
Influenza A viruses isolated from wild bird (overwintering duck) samples collected in Guatemala between 2010 and 2013. The color-coded genome constellations shown for each virus were determined based on phylogenetic clades with bootstrap support of >70%. Color notations for each gene segment (columns) are independent of each other. Each shade of blue represents a different clade within the main North American lineage, orange represents the most divergent North American lineage, and green represents the Eurasian lineage. Different subtypes of HA and NA are shown on the right. Asterisks in the strain name denote mixed infections. AMWI, American wigeon; BWT, blue-winged teal; GWT, green-winged teal; NOSH, northern shoveler.

To further analyze the virus diversity, we determined the genome constellations (i.e., specific combination of segments for each virus, sometimes referred as a genotype) of each of the 68 IAV isolates. For this analysis, each viral segment was assigned to its corresponding lineage (North American, Eurasian, etc.). Since the majority of gene segments fell within the main North American lineage, these segments were further classified into clades supported by >70% bootstrap value (see Fig. S1 to S3 for the position of the clades in the phylogenetic trees). This analysis revealed an array of different genome constellations that included 26 different combinations of internal gene segments and 52 total combinations when the HA and NA subtype was included (Fig. 2). Overall, the genome constellations varied among and within subtypes. Similar levels of diversity were found throughout each sampling period without any discernible patterns, consistent with widespread reassortment among LPAIVs in nature (21). Notably, gene segments from divergent lineages not previously detected in Guatemala, and rarely detected in the United States, were observed for the PA, NP, and NS segments as detailed below.

### LPAIVs found in overwintering ducks in Guatemala are positioned outside major genetic lineages (PA, NP, and NS segments).

For the PA gene segment, two viruses recovered during the 2012-2013 migration in Guatemala clustered within the Eurasian lineage, along with other recent viruses from the United States collected during 2006 and 2013 (Fig. 1A). These viruses were A/blue-winged teal/Guatemala/CIP049-14/2011 (H3N3) and A/blue-winged teal/Guatemala/CIP049H114-76/2013 (mixed) and were isolated from samples collected in November 2012 and January 2013, respectively. Their internal gene segments were positioned in different subclades in the phylogenetic trees, indicating reassortment with other viruses. The introduction of this clade of Eurasian viruses has been previously documented in Alaska (26) and in California, albeit with limited circulation (25). This Eurasian-to-North America introduction occurred recently and is unrelated to the major incursion of Eurasian genes that occurred in the 1960s that gave rise to one of the two main North American PA lineages (27, 28). Identification of this lineage in Guatemala indicates that its geographical spread has continued to Central America. A time-scaled maximum clade credibility (MCC) tree, including background Eurasian PA sequences and related sequences from North America, estimates that the introduction of these reassortant viruses occurred between 2007.1 and 2010.4 (95% highest posterior density [HPD]) (Fig. S4).

10.1128/mSphere.00362-16.4 FIG S4  Maximum credibility trees for PA, NP, and NS allele A gene segments. The stars indicate the minor divergent lineages from which viruses were found in Guatemala. Color annotations are the same as those in Fig. S1. The posterior probability and 95% HPD for each node date are indicated. Download FIG S4, PDF file, 1.4 MB. Copyright © 2017 Gonzalez-Reiche et al. 2017 Gonzalez-Reiche et al. This content is distributed under the terms of the Creative Commons Attribution 4.0 International license.

For the NP and NS gene segments, five viruses, also recovered during 2012 to 2013, clustered with viruses from the United States from 2007 in a separate minor lineage that is basal to the major North American lineage (Fig. 1B; see also Fig. S3). For both genes, this lineage has been described as an “old western-avian lineage,” with apparent low prevalence in North America (28). BLAST searches for these viruses resulted in only 21 matching viruses from the United States between 1987 and 2014, ~0.3% of over 6,000 NP sequences of avian North American viruses available in GenBank from that time period, with >90% identity (percent identity level that split the viruses into main genetic lineages in the phylogenetic trees). Similarly, only 23 matches with North American viruses from 1987 to 2014 were found for NS (~0.4% of >6,000 North American avian virus NS sequences), with shared identities of >90%. Interestingly, although still within the main North American lineage, other internal gene segments of these five divergent viruses (PB2, PB1, PA, and M) consistently clustered together with bootstrap support values of >90% (PB2, PB1, and M) with the same most divergent North American strains (Fig. S1 to S3). In agreement with a previous study (28), the estimated time of divergence of these lineage was about 100 years ago in inferred time-scaled MCC trees for NP (1861.4 to 1922.6, 95% HPD) and NS (1913.1 to 1941.1, 95% HPD), including all the closest BLAST matches and additional background avian viruses representing the global diversity (Fig. S4).

### The HA and NA subtypes of the LPAIVs found in Guatemala are genetically diverse.

The subtype of the two surface glycoprotein genes (HA and NA) was identified through BLAST searches (29). In all cases, the top matches were contemporary viruses (from 2009 to 2013) from North America, including the subtypes H1 to H5, H7, H11, H12, H14, N2 to N6, and N8 (24). Comparison of the viruses from Guatemala through nucleotide pairwise distance revealed that despite their high identity (>95%) with North American strains, the five viruses with divergent internal gene segments also had highly divergent surface glycoprotein genes of the subtypes H3N8 (*n* = 2) and H4N2 (*n* = 3). For the two H3N8 genes, the H3 variants shared up to 100% identity with each other but only 83% with the other H3 genes from Guatemala. The two N8 genes shared 98.7% identity with each other but only 79% with the other N8 genes isolated in Guatemala. Similarly, the H4N2 H4 variants shared only 84% identity with the other Guatemalan H4s, whereas the N2 variants shared only 87% identity with the other Guatemalan N2 genes (Fig. S5). In GenBank, less than 0.9% of H3 (*n* = 10), 0.6% of H4 (*n* = 9), 0.5% of N2 (*n* = 6), and 0.6% of N8 (*n* = 8) avian IAV sequences reported for North America were highly similar to these viruses (>95% identity).

10.1128/mSphere.00362-16.5 FIG S5  Pairwise percent identity matrices for H3, H4, N2, and N8 nucleotide sequences of LPAIV isolates recovered from overwintering waterfowl in Guatemala, 2010 to 2013. The levels of similarity between each pair of viruses are indicated by the colors, where the darkest shade of orange represents the highest similarity and yellow represents the lowest. Download FIG S5, PDF file, 0.3 MB. Copyright © 2017 Gonzalez-Reiche et al. 2017 Gonzalez-Reiche et al. This content is distributed under the terms of the Creative Commons Attribution 4.0 International license.

**FIG 3:**
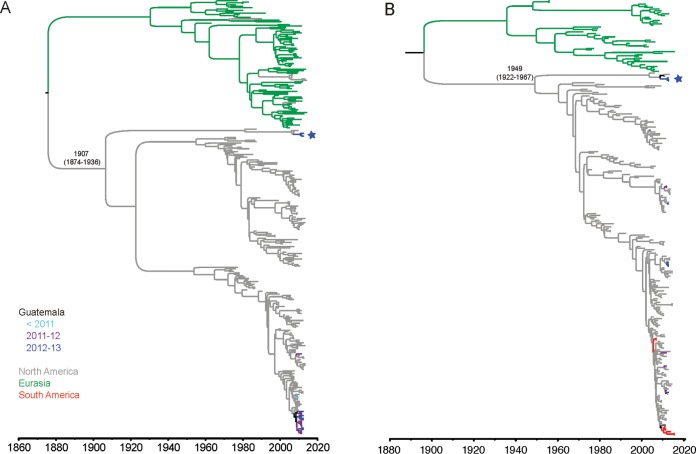
Phylogenetic relationships of H3 and H4 segments. Time-scaled MCC trees inferred for H3 genes of LPAIV from Guatemala and background global sequences from 1963 to 2014 (A) and H4 genes of LPAIV from Guatemala and background global sequences from 1956 to 2014 (B). The TMRCAs between the most divergent lineage and the main North American lineage and their corresponding 95% HPD intervals are shown. The divergent lineages found in Guatemala are indicated by the stars.

For the H3, H4, N2, and N8 glycoprotein genes, time-scaled phylogenetic trees using the BEAST program (30) with global background viruses were inferred to estimate the time of divergence of the basal lineage. For H3, the basal lineage diverged from the main North American lineage approximately a century ago (1874.2 to 1936.5, 95% HPD) (Fig. 3A; see also Fig. S4). The estimated time of divergence between the H4 basal lineage and the main North American lineage was over 50 years (1922.5 to 1966.7, 95% HPD) between the two lineages (Fig. 3B; see also Fig. S4). For N8, the estimated time to the most recent common ancestor (tMRCA) between the basal lineage and the main North American lineage was over 100 years ago (1838.5 to 1933.1, 95% HPD). For N2, the viruses clustered on a monophyletic clade within the main North American lineage with an estimated time of divergence of over 40 years (1971.0 to 1973.8, 95% HPD) (Fig. S6).

10.1128/mSphere.00362-16.6 FIG S6  Maximum clade credibility trees for the H3, H4, N2, and N8 glycoprotein genes and the PA gene. Color annotations correspond to those indicated in Fig. S1. Branch lengths are in years. The posterior probability and 95% confidence interval (CI) are indicated for each node date. The stars indicate the minor divergent lineages. Download FIG S6, PDF file, 2.2 MB. Copyright © 2017 Gonzalez-Reiche et al. 2017 Gonzalez-Reiche et al. This content is distributed under the terms of the Creative Commons Attribution 4.0 International license.

**FIG 4:**
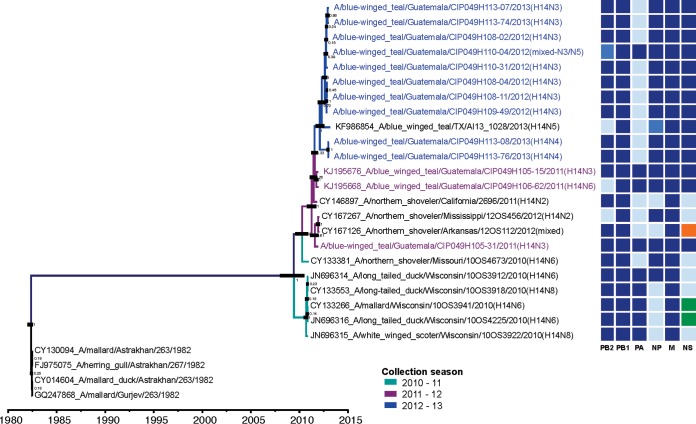
Diversity of avian influenza virus H14 subtype. The maximum clade credibility tree for the HA gene is shown. Branches are colored according to migration seasons of 2010 to 2011, 2011 to 2012, and 2012 to 2013. The taxon names for the viruses from Guatemala are shown in colors according to the respective collection seasons. The color-coded genome constellations for the internal genes shown on the right were determined based on phylogenetic clades with bootstrap support of >70%. Color notations for each gene segment (columns) are independent of each other. Each shade of blue represents a different clade within the main North American lineage, orange represents the most divergent North American lineage, and green represents the Eurasian lineage. Thirteen out of 14 virus isolates from Guatemala of the H14 subtype were included; the genome sequence of the isolate A/blue-winged teal/Guatemala/CIP049H109-76/2012 (H14N3) was not available at the time of performing the analysis.

In addition to detecting viruses with H3 and H4 segments that are divergent from the main North American lineage, an unexpectedly large number (*n* = 14) of H14 subtype viruses were identified in overwintering blue-winged teals in Guatemala (24, 31). H14 is a subtype of Eurasian origin and was detected in the Western Hemisphere only within the last decade and only at a low prevalence (32–34). During the 2012-2013 season, we detected the largest number of H14 subtype viruses ever reported in dabbling ducks (the same number of virus sequences available globally for this subtype by 2013, *n* = 14), specifically in blue-winged teals (24). Time-scaled MCC trees inferred for all H14 sequences available up to 2013 showed clustering of the H14 HA genes from Guatemala by season, interspersed with North American viruses (Fig. 4). These results indicate that the H14 viruses, instead of persisting locally in Guatemala between migrations, were independently introduced to overwintering ducks in Guatemala each season during 2011 to 2012 and 2012 to 2013. This observation also is supported by the lack of similarity among the internal gene constellations of the Guatemalan H14 viruses compared with North American isolates and between overwintering seasons (Fig. 4).

### Multiple introductions contribute to viral genetic diversity in overwintering ducks in Guatemala.

**FIG 5:**
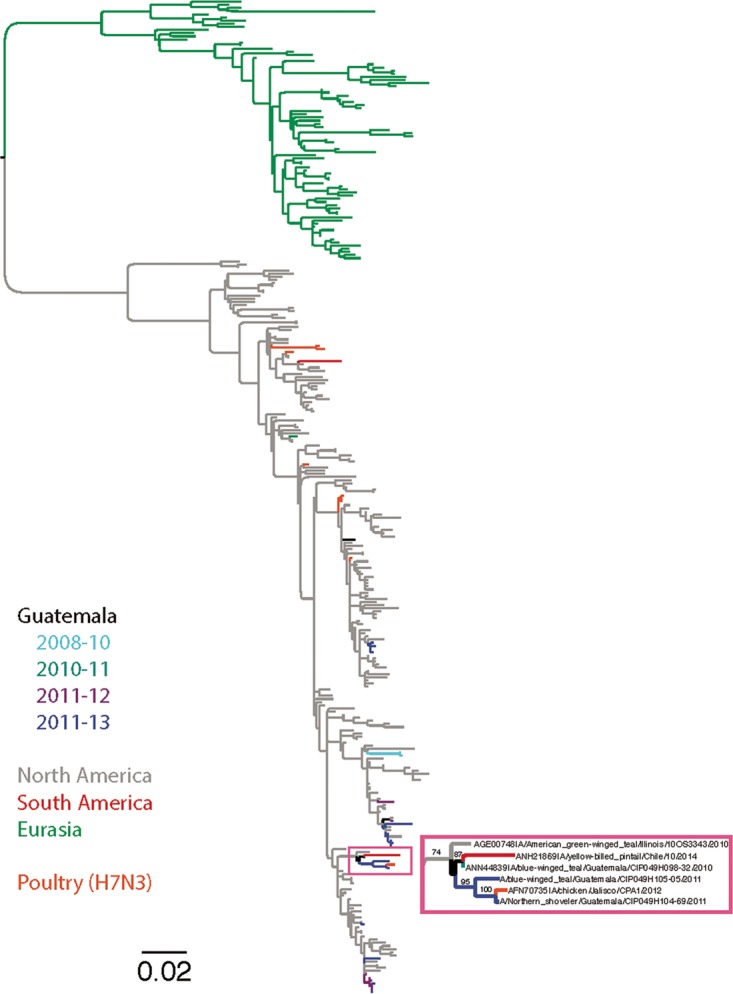
Phylogenetic relationships of the N3 gene segment. Phylogeny inferred using maximum likelihood method of N3 NA of LPAIV from Guatemala and other global viruses collected between 1975 and 2014. Viruses are shaded by geographic location. The tree is midpoint rooted, and all branch lengths are drawn to scale. The branch with the viruses from Guatemala and the HPAIV H7N3 from Mexico is delineated by the pink box and shown on the left.

At overwintering sites, overlap of migrating birds with resident bird populations may result in virus spillover from one population to another (18, 35). Given the diversity of genome constellations observed, we analyzed whether this diversity is the result of multiple introductions and if locally persisting viruses in Guatemala could be detected in overwintering ducks. We inferred global ML phylogenies of the most abundant glycoprotein subtypes (H3 [*n* = 22], H4 [*n* = 13], and N3 [*n* = 21]) and searched for monophyletic gene clusters exclusive to viruses recovered in Guatemala over multiple years, indicative of virus persistence over time. For the H3 subtype, two introductions were detected, and although most viruses grouped together (*n* = 17 of 22), the cluster was not limited to viruses detected in Guatemala. For the H4 subtype, the viruses were segregated by season of detection, in seven different positions across the main North American and minor basal lineages, representing independent virus introductions. For N3, there were four separate introductions segregated by season of detection, but none of these supported introduction of locally persisting viruses (detailed global phylogenies are shown in Fig. S7). Interestingly, three of the N3 viruses isolated in Guatemala (one from 2010 and two from 2011) were the closest relatives (with bootstrap support values of >80%) of the HPAI H7N3 strain isolated from poultry in Mexico in 2012 (11) (Fig. 5), which also clustered with two North American-origin viruses recently isolated in South America. In summary, for the subtypes analyzed and during 2010 to 2013, we detected multiple virus introductions without evidence of locally persisting viruses spreading to overwintering ducks in Guatemala.

10.1128/mSphere.00362-16.7 FIG S7  Global maximum likelihood phylogenetic inference of the H3 and H4 hemagglutinin and the N3 neuraminidase gene segments. Bootstrap support values are shown as branch labels. The Eurasian (green) and the Oceania (fuchsia) lineages are collapsed for easier representation. The “gull” lineage is also collapsed on the N3 phylogeny. The stars indicate the positions of the separate virus introductions found in Guatemala. Color annotations are the same as those for Fig. S1. Download FIG S7, PDF file, 2.5 MB. Copyright © 2017 Gonzalez-Reiche et al. 2017 Gonzalez-Reiche et al. This content is distributed under the terms of the Creative Commons Attribution 4.0 International license.

## DISCUSSION

The observation that the genetic diversity of LPAIV is higher in areas where migration flyways overlap and duck populations congregate (17, 36–39) helps to target surveillance efforts toward key source populations. Similarly, we find that ducks overwintering in Guatemala harbor relatively high genetic diversity and elevated detection rates (24). This diversity is reflected in the number of genome constellations that were observed (52 different combinations from 68 recovered viruses). An unexpected finding, considering that most of the virus isolates came from only one species (blue-winged teals), is that with the exception of the shorebird lineages, we detected viruses from most of the contemporary clades that circulate in North American wild waterfowl, including a rare divergent lineage. Waterfowl species that migrate to Central America will usually follow more than one migration flyway to and from their breeding grounds (40). In agreement with this behavior, a recent study suggests that introduction of LPAIVs from breeding to wintering sites and vice versa is likely to occur from different regions and is not restricted to a single flyway (19). Although the blue-winged teals migrate mainly through the Central and Mississippi flyways, at their stopover or wintering sites they overlap other species (e.g., northern shovelers, northern pintails, and green-winged teals) that migrate through different routes, such as the Pacific or Atlantic flyway (40–43). The high viral diversity observed in blue-winged teals in Guatemala supports the notion that congregations of birds overwintering in Central America increase interspecies virus transmission, virus diversity, and opportunities for coinfection and reassortment. The small sample size and the bias introduced by opportunistic sampling of hunted ducks currently limit analysis of virus coinfection and reassortment. Although we were able to characterize multiple variants of surface glycoprotein and internal gene segments present in mixed infections in 16% of the samples, the prevalence of mixed infections in overwintering birds in Guatemala and how it compares to other locations remain unclear. In addition, *in vitro* propagation of IAV, such as virus isolation in embryonated chicken eggs, imposes a strong selective bottleneck reducing the number of variants present on a field sample (44, 45); consequently, the percentage of mixed infections observed in this study is most likely an underestimate.

An important outstanding question is why so many rare genetic lineages of surface glycoproteins (H14 and divergent H4, H3, N2, and N8), and unusual subtype combinations were identified in blue-winged teals overwintering in Guatemala (23, 24) and how this diversity relates to variations over time in heterosubtypic immunity in the host population (46, 47). The sources of this diversity also remain unclear. The divergent viruses were recovered from blue-winged teals, a northern shoveler, and an American wigeon and, with the exception of H14, had the same genome constellations as each other but different from those of other North American viruses. A virus from the same lineage was detected in Louisiana in 2013 (48). Comparably, the H14 subtype has been only sporadically found in North America, with low seroprevalence (34). Given the lack of evidence for the sustained circulation of these unusual viruses in North America, we speculate that these rare subtypes have been introduced to Guatemala from an unknown source. In Guatemala, quantitative reverse transcription PCR (qRT-PCR)-positive samples obtained from year-long-resident species, such as black-bellied whistling duck, fulvous whistling duck, and American coot, have resulted in unsuccessful virus isolation attempts (24). However, virus isolates have been recovered in other tropical locations (9, 49), from these and other species that also reside in Guatemala. Intensive sampling of these potential avian reservoirs, with a geographical range more restricted to the Neotropics, as well as intensive sampling of other migratory species (in addition to hunted birds and different from the blue-winged teal), might help to uncover additional sources of the viral genetic diversity observed in Guatemala.

Avian influenza surveillance in Guatemala was originally established for the timely detection of HPAI strains, including Eurasian-origin H5N1 viruses (23). In this study, detection of genes from the Eurasian lineage, in addition to the H14 subtype, demonstrates that virus spread of Eurasian strains by migratory birds to Central America is plausible over the course of only a few migration cycles. This finding is important considering the recent introduction of Eurasian HPAI in North America (12) and its potential implications for Latin America. On this note, the detection of N3 genes closely related to the HPAI H7N3 strain that caused a poultry outbreak in Mexico in 2012 underscores the significance of increasing surveillance at both ends of the migration flyways. Last, we did not detect reassortant strains of the South American lineage. This result may be a consequence of the small sample size and limited number of sampled host species. The gap in avian IAV surveillance in countries from Latin America is still significant, making it hard to explain the lack of recovery of viruses from South American lineages in the presence of closely related viruses of North American origin in Guatemala and South America (internal gene segments and N3 subtype).

In summary, our results highlight the importance of including understudied geographic locations to unveil the total diversity of circulating strains in the Western Hemisphere. To our knowledge, the genomes reported in this study constitute the greatest collection of LPAIVs from Central America. Our observations offer additional evidence that stopover habitats and overwintering sites with overlapping flyways serve as places for virus transmission, linking host populations from different locations through genetic reassortment. We propose that the existence of a natural geographical corridor in Central America provides such a niche for birds migrating from North America, but we recognize that year-round surveillance that includes resident bird populations and additional locations in Central America is needed to understand the ecology of IAV in this region.

## MATERIALS AND METHODS

### Definitions.

Throughout this article, the United Nations geographical region and subregion classifications for North, Central, and South America and Latin America were used (50).

### Virus full-genome sequencing.

The methods for collection and isolation of the 68 viruses has been previously described (24). For genome sequencing, 45 genomes were first sequenced in a 454 GS Junior system as described before (31), with one modification. For 30 of the 45 sequenced genomes, a multisegment amplification of IAV genes (MS-RTPCR) (51) was performed. Sequencing reads were processed for a shotgun run with the Junior Newbler software (v.2.7 and 3.0; Roche, Branford, CT). First, the contigs were assembled *de novo* with the program GS De Novo Assembler v.3.0, and BLAST searches were performed to identify the closest matches. Reference genomes were then obtained from the NCBI GenBank database and used to reassemble the contigs using the GS Reference Mapper software (v.3.0). Genome assembly was refined after several iterations of reference mapping to resolve mixed infections (when present); for the internal gene segments, this was achieved by including two or more reference sequences with sequence identity of <97% for the “mixed” segment and adjusting kmer size in the alignment parameters. The remaining viruses (*n* = 23) were sequenced by high-throughput Illumina sequencing as previously described (52). For this set of genomes, it was not possible to resolve individual gene variants for the internal gene segments; thus, mixed infections were identified by observing a high frequency (>10%) of single nucleotide variants across the entire length of one or more gene segments.

### Maximum likelihood phylogenetic inferences for internal gene segments.

For all internal gene segments (PB2, PB1, PA, NP, M, and NS), background virus strains with full-genome sequence data, collected between 2000 and 2013 from waterfowl species from different intercontinental genetic lineages available at the Influenza Research Database (IRD) (53), were included in the analysis. All sequence alignments were done with MUSCLE (54) as integrated in the program MEGA 6.0 (55). The best-fit model of nucleotide substitution was determined for each gene using the Bayesian information criterion (BIC). Final phylogenetic trees were constructed using ML inference with the general time-reversible (GTR)+G (PB2, PB1, PA, NP, and NS) or GTR+G+I (NS) nucleotide substitution model. Robustness of tree topologies was assessed with 100 neighbor-joining bootstrap replicates.

### Analysis of genome constellations.

The genome constellations of the viruses from Guatemala were determined by classifying the viruses in the main lineages observed in the ML phylogenetic trees. The lineages were further subdivided in clades supported by >70% bootstrap values. Clades were defined independently for all internal gene segments. For the surface glycoprotein genes (HA and NA), searches with full-length nucleotide sequences were performed in BLAST to identify the top 10 hits. In addition, pairwise distances were computed and isolates of the same subtype with <90% identity to each other were used to identify divergent variants. Genome constellations were defined as unique gene combinations at the level of clades; colors were assigned to different clades and used to build graphical representations.

### Phylogenetic analysis of the surface glycoprotein genes.

For the surface glycoprotein genes, phylogenies were inferred for H3, H4, and N3. To infer global ML phylogenies, all avian full-length coding sequences available for these subtypes in IRD were included in the analyses (H3, *n* = 1,266; H4, *n* = 1,054; and N3, *n* = 833 global sequences). The trees were inferred using RAxML v8.2.4 under the GTRCAT model of nucleotide substitution. Robustness of tree topologies was assessed with 500 bootstrap replicates. For N3, we reduced the number of background sequences, ensuring maintenance of representation of the main lineages observed in the global tree, except for the most divergent “gull” lineage. The reduced ML tree (*n* = 318 sequences) was built under the best-fit HKY+G model of nucleotide substitution in MEGA 6.0 and assessed with 500 bootstrap replicates.

### Time-scaled phylogenetic analysis.

Time-scaled phylogenies for H3, H4, N2, and N8 were inferred with background sequences representative of all lineages observed in the ML phylogenies of these genes. New ML phylogenies were inferred, and the trees were inspected in the program Path-O-Gen to identify outlier sequences. These sequences were then excluded from the analysis to infer time-scaled phylogenies in the BEAST package v.1.8.2 (30). For H3, the dated phylogeny was inferred under the GTR model of nucleotide substitution, with a relaxed uncorrelated lognormal (UCLN) molecular clock and constant population size. A prior of 0.001 was used for the mean rate as estimated by previous analysis of the data with the program Path-O-Gen (56). For H4, a Bayesian skyline coalescent tree prior was used with the uncorrelated exponential relaxed clock model as previously described (27). The phylogenies for NP, NS, N2, and N8 were inferred under the stasis and rate-shift diversification (SRD) model with the UCLN molecular clock, at a constant population size, with a mean rate prior of 0.001. For the PA protein-encoding segment, a total of 157 taxa were included, and the MCC tree was inferred under the GRT+G+I model. The coalescent model with constant population was used as tree model, with a relaxed lognormal clock model. For the mean rate, a prior of 0.002 was used (57). For all data sets, the Monte Carlo Markov chain (MCMC) was run in triplicate for 50 to 100 million generations. For the H14 subtype, a phylogenetic tree was initially inferred using the minimum evolution method under the Tajima-Nei best-fit model of nucleotide substitution with gamma distribution (TN93+G). All available sequences of the H14 subtype from North America and Europe were included. A time-scaled phylogeny was inferred after comparing different clock models, using the SRD06 substitution model with relaxed exponential clock model. A coalescent exponential growth was used as tree model. A prior of 0.001 was used. The analysis was done in triplicate for 10 million generations/replicate to ensure convergence. For all analyses, the program Tracer (v.1.6.0) was employed to observe convergence of parameters. The results of all replicates of the same gene segment were combined after removal of the burn-in of 10 to 15% using LogCombiner (v.1.8.2). The MCC trees were reconstructed in TreeAnnotator (v.1.8.2) and visualized and annotated in the FigTree program (v.1.4.2).

### Accession number(s).

For all genomes, final consensus sequences were submitted to GenBank (accession numbers KX365054 to KX365077, KX960368 to KX960493, KX986855 to KX986872, KY644193 to KY644513, KY653928 and KY653929, and KY673152 to KY673176).
