# Supplementary figures and images for: Evidence of Intercontinental Spread and Uncommon Variants of Low-Pathogenicity Avian Influenza Viruses in Ducks Overwintering in Guatemala
Source: mSphere. 2017 Apr 5;2(2):e00362-16. doi: 10.1128/mSphere.00362-16 (PMC5381266; doi:10.1128/mSphere.00362-16)

PB2

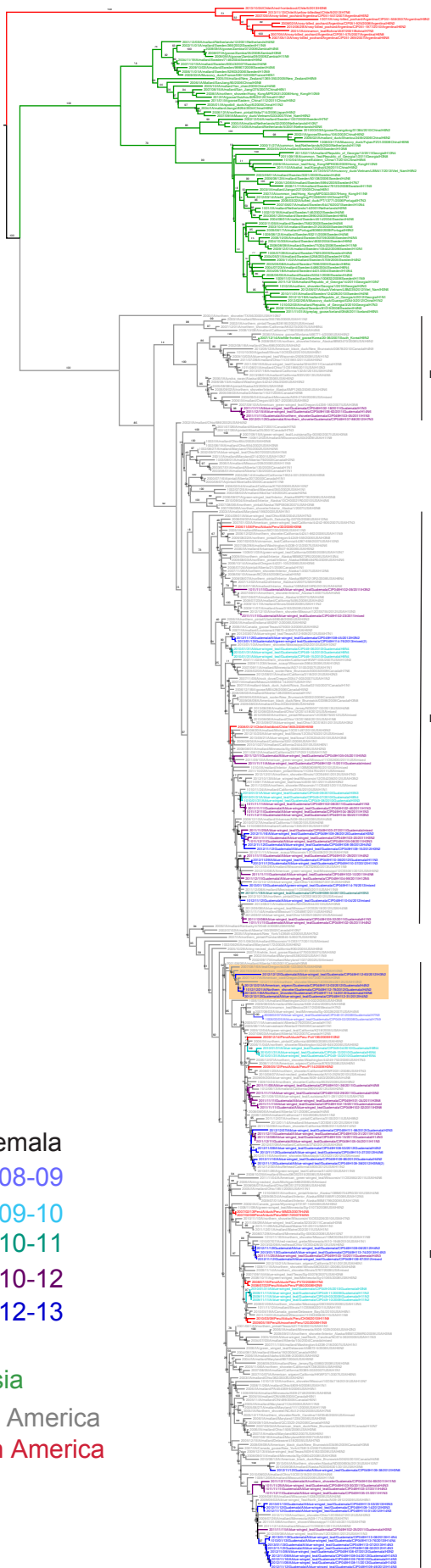

PB1

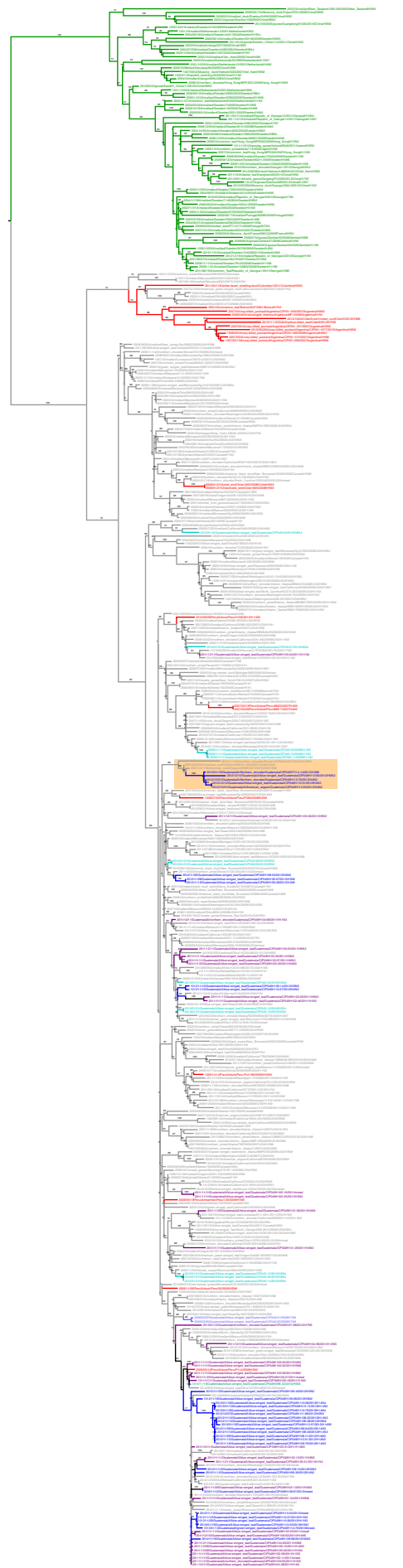

Guatemala  
2008-09  
2009-10  
2010-11  
2010-12  
2012-13

Eurasia  
North America  
South America

0.03

0.01

Supplement: FIG S1 [file sph002172264sf1.pdf]

PA

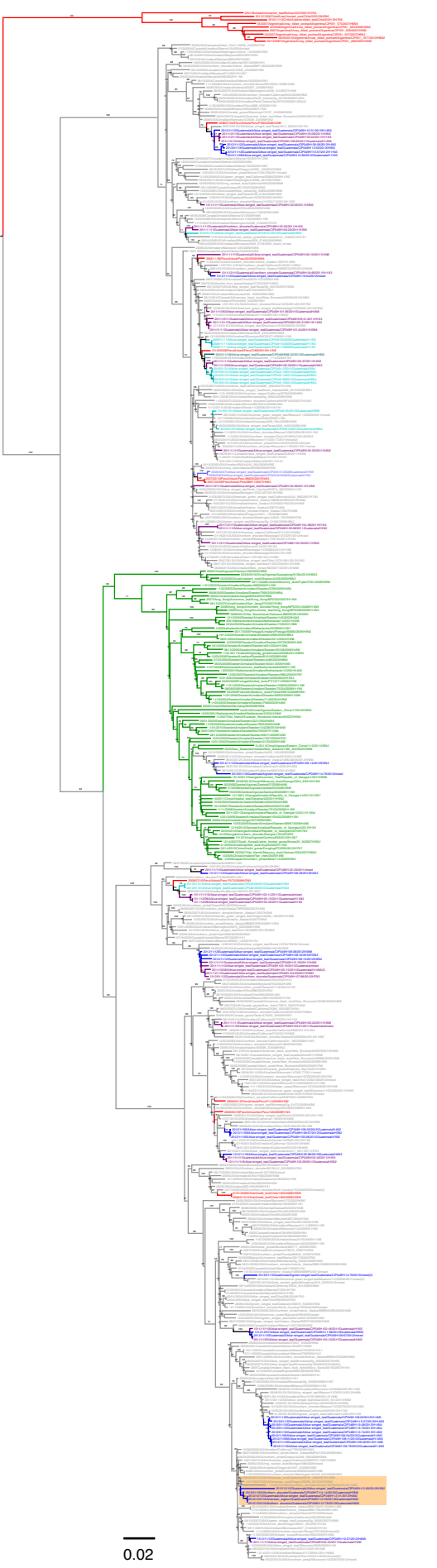

NP

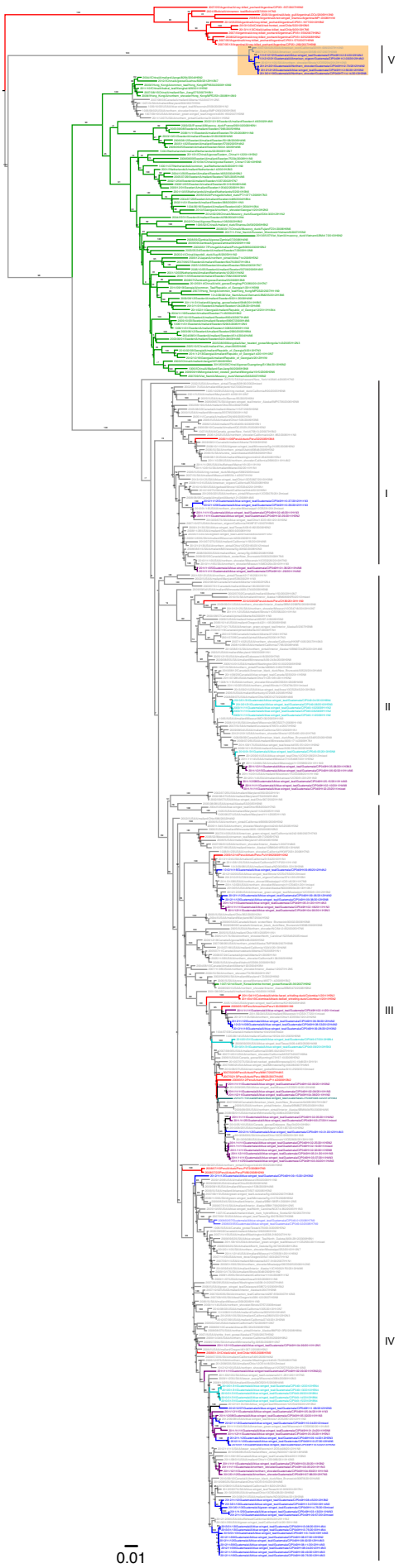

Supplement: FIG S2 [file sph002172264sf2.pdf]

M

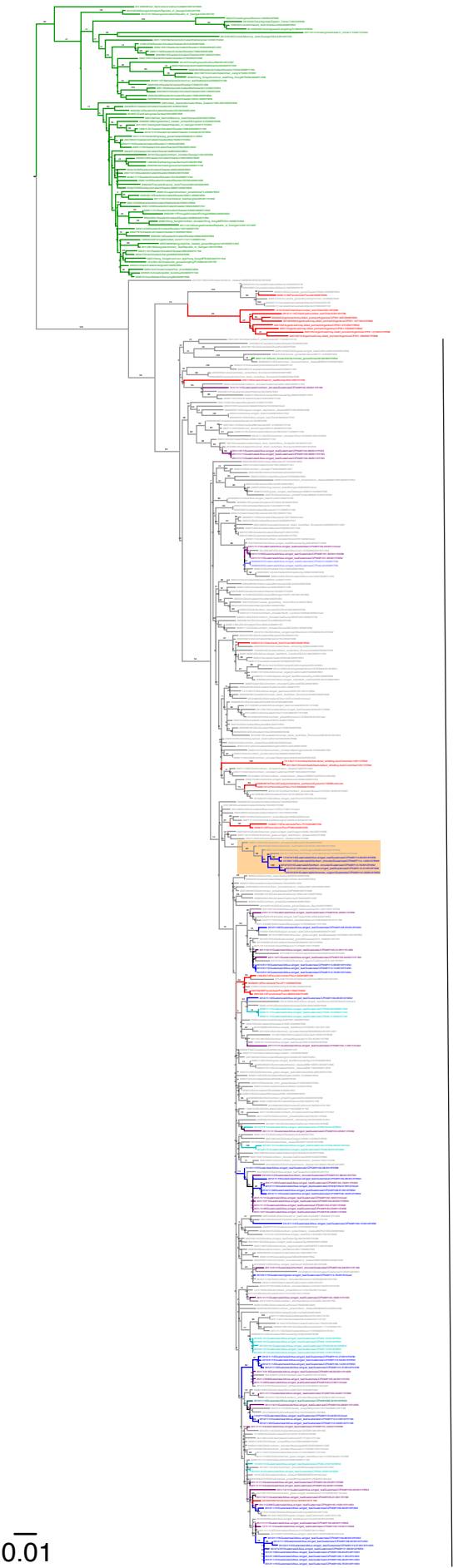

NS

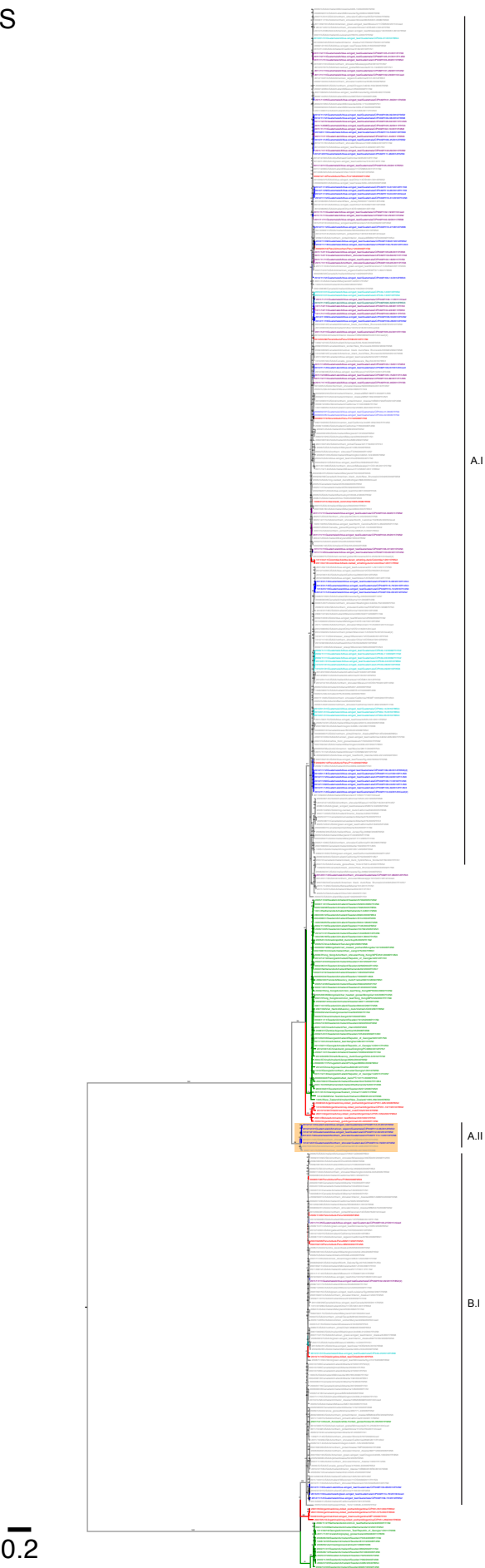

Supplement: FIG S3 [file sph002172264sf3.pdf]

PA

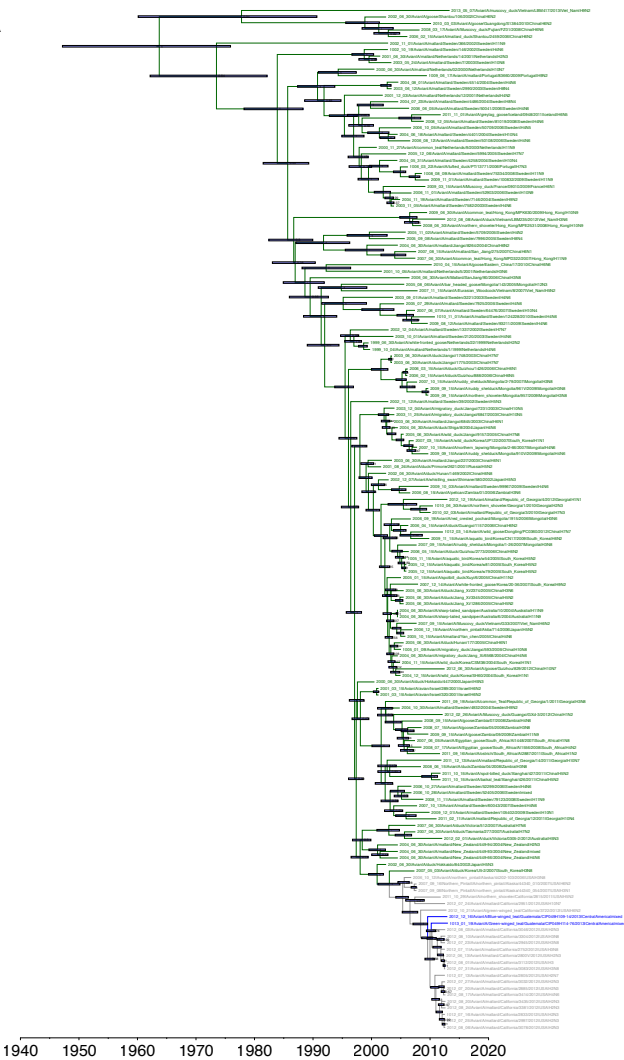

NP

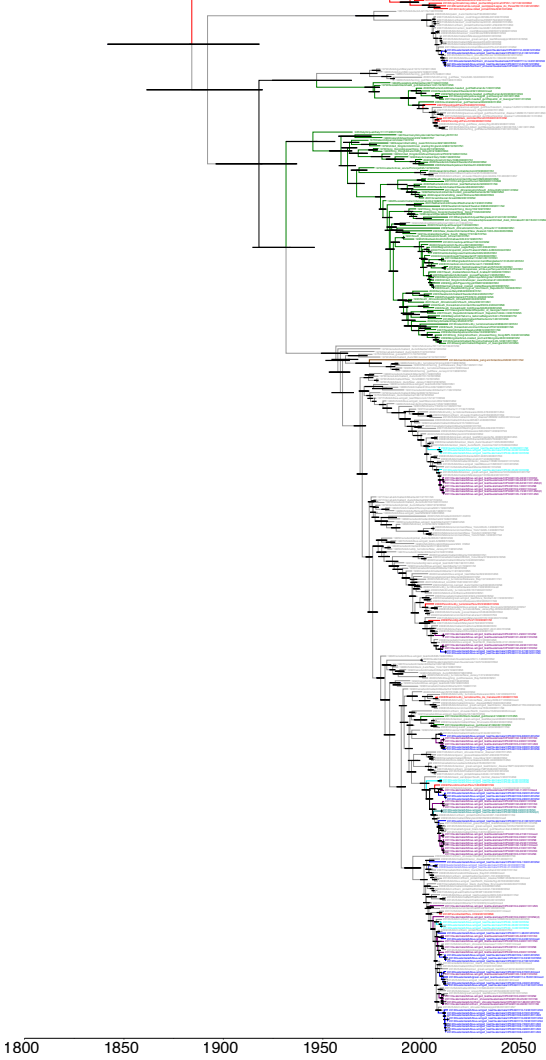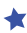

NS allele A

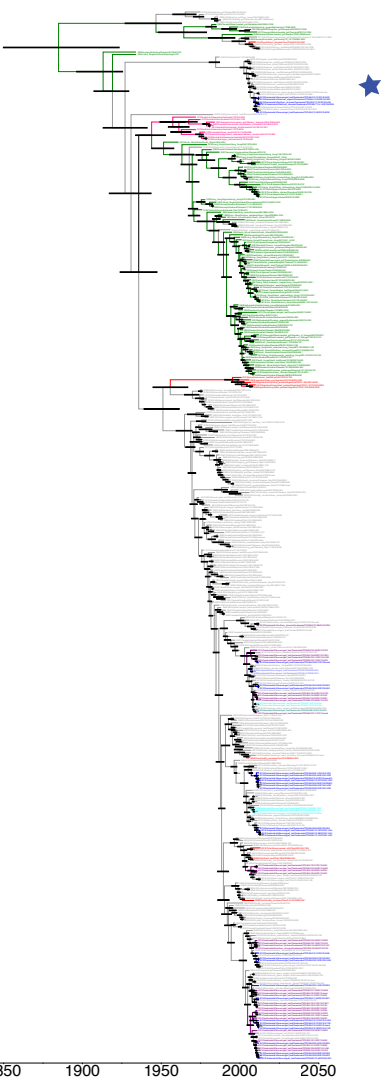

Supplement: FIG S4 [file sph002172264sf4.pdf]

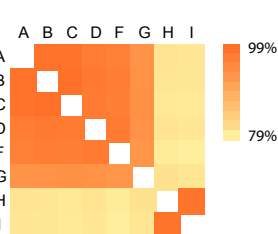

Supplement: FIG S5 [file sph002172264sf5.pdf]

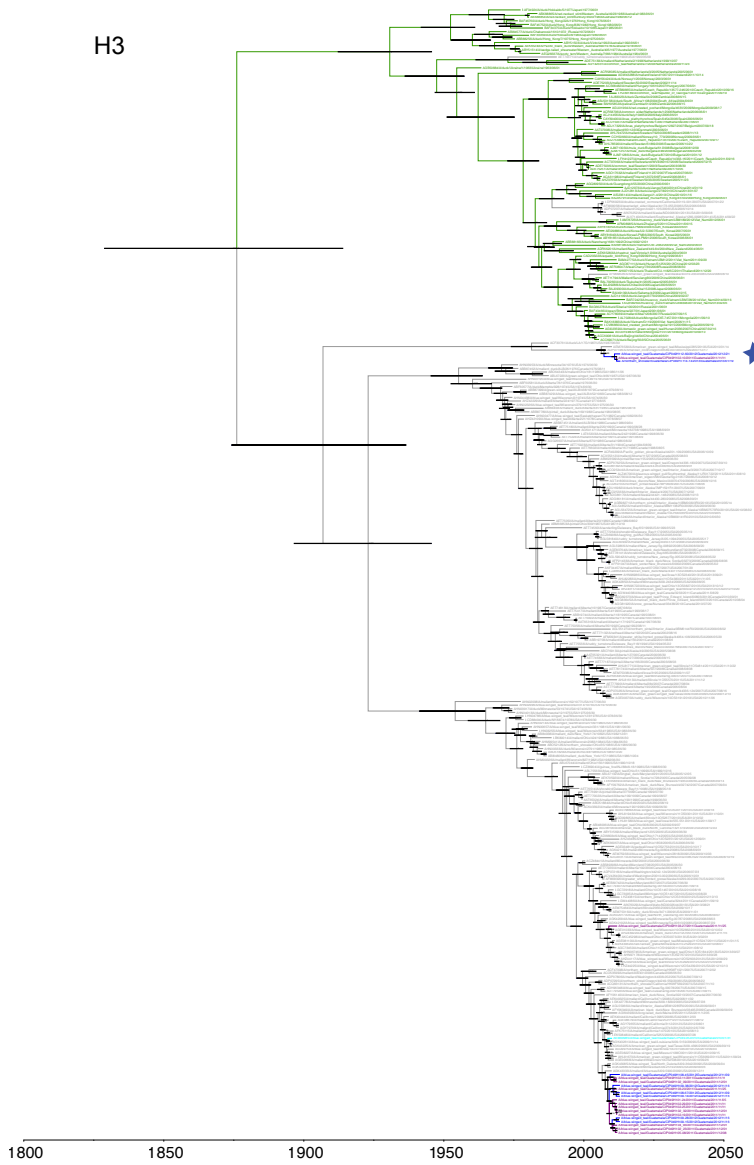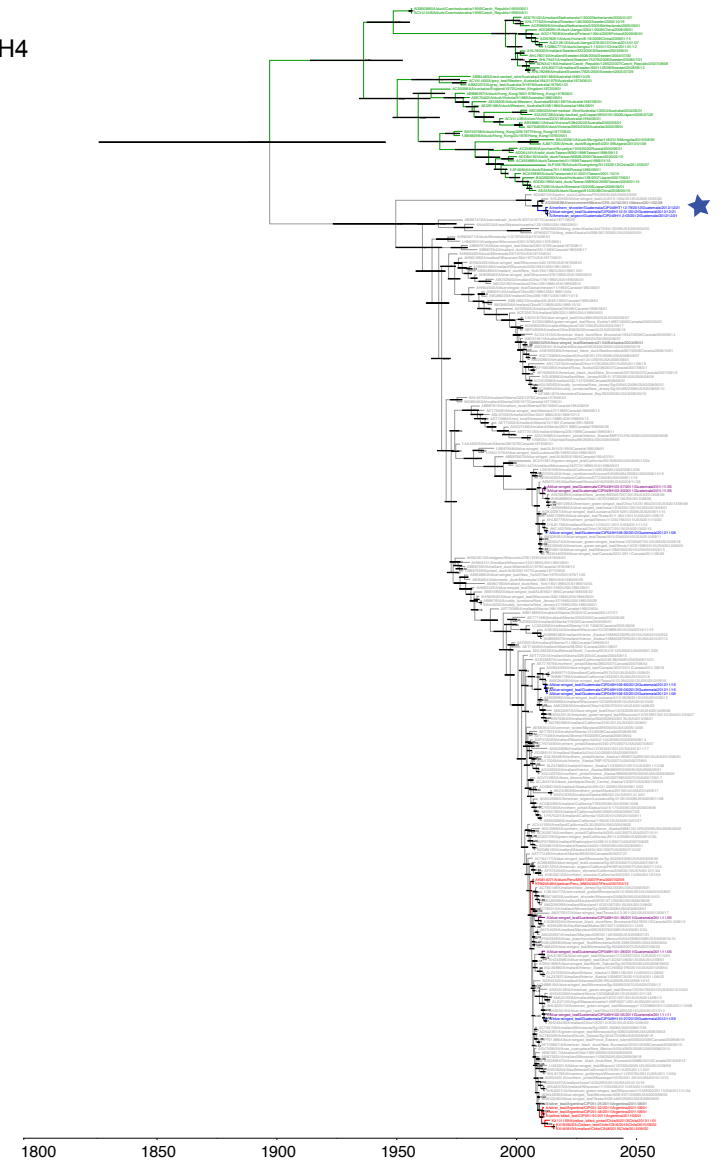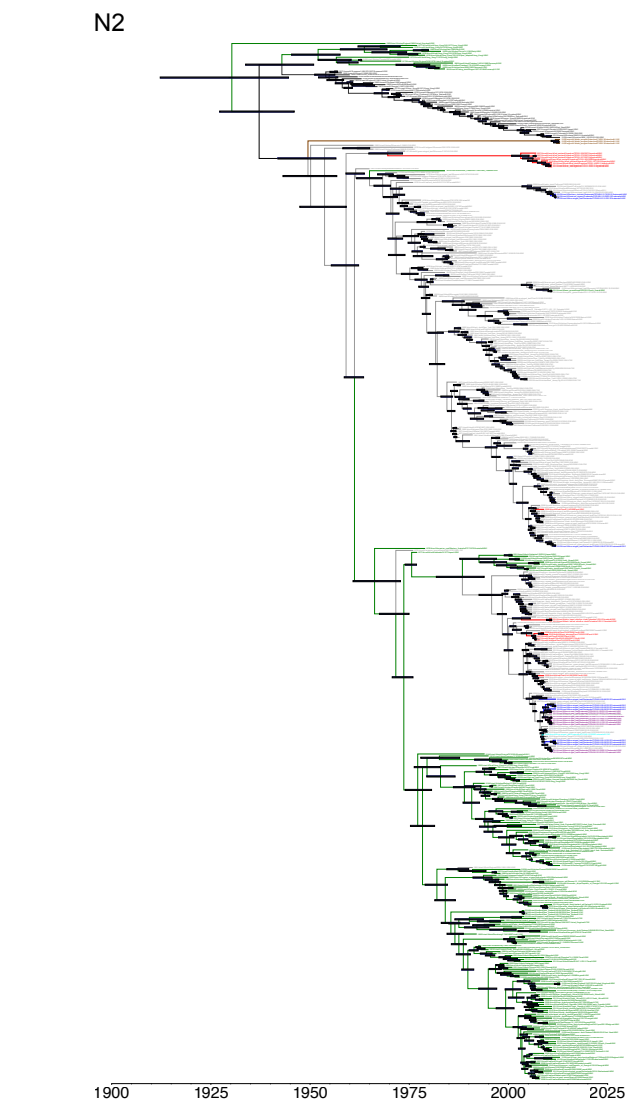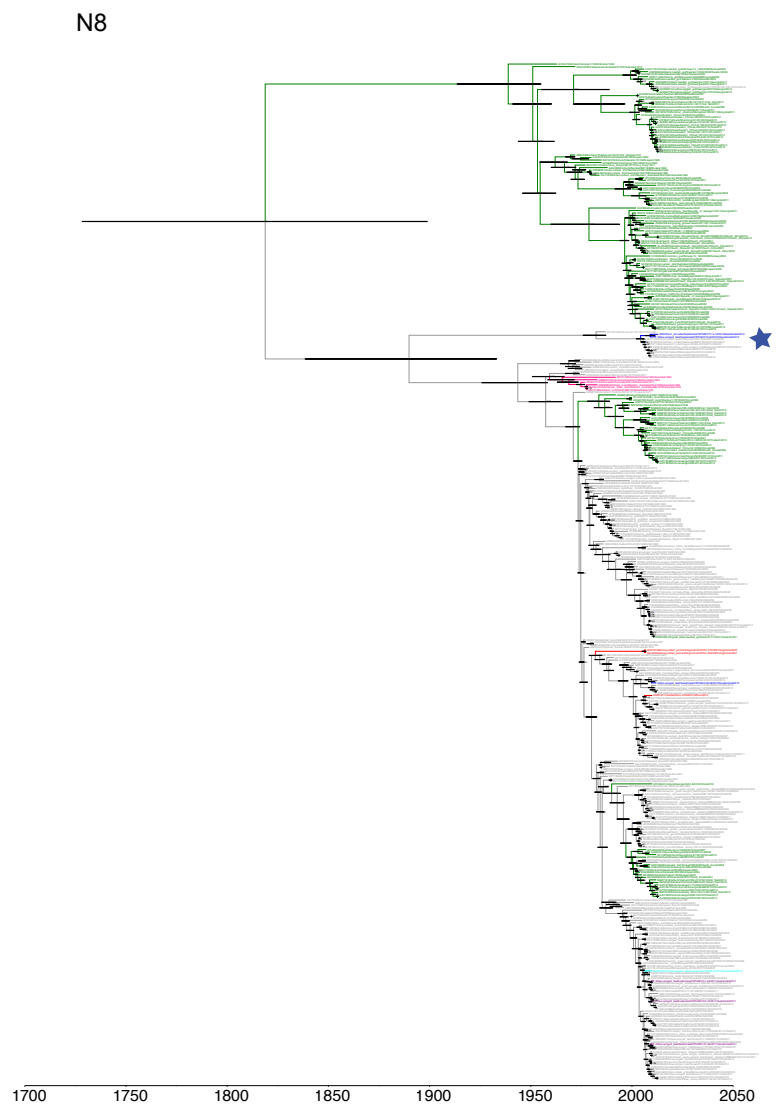

Supplement: FIG S6 [file sph002172264sf6.pdf]

H3

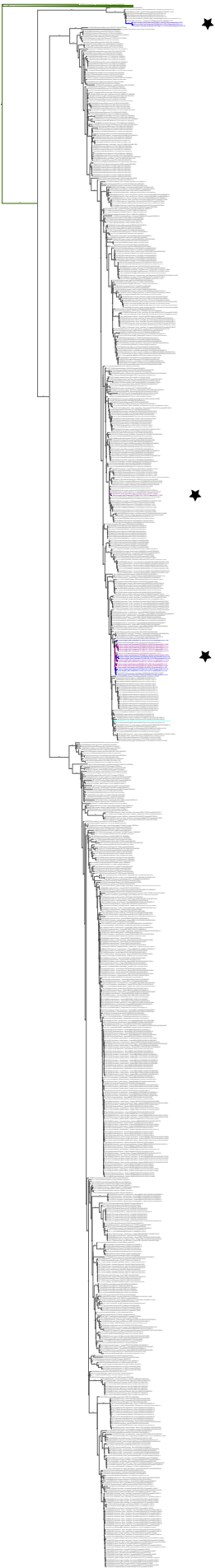

H4

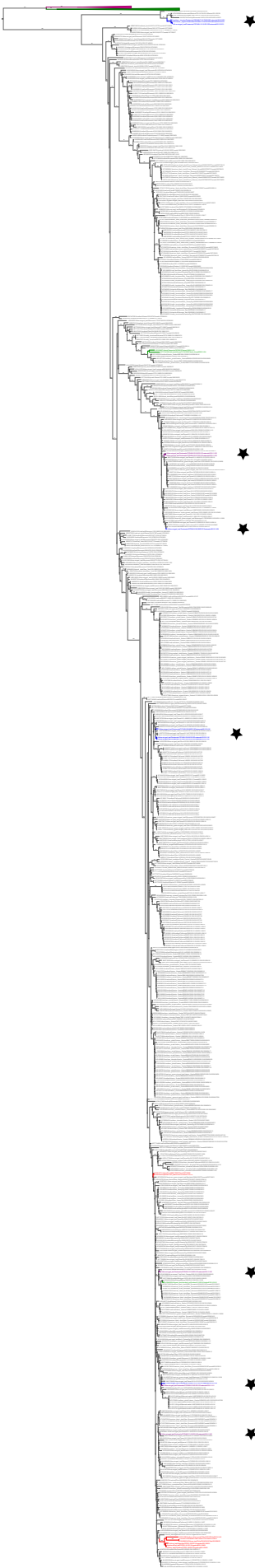

N3

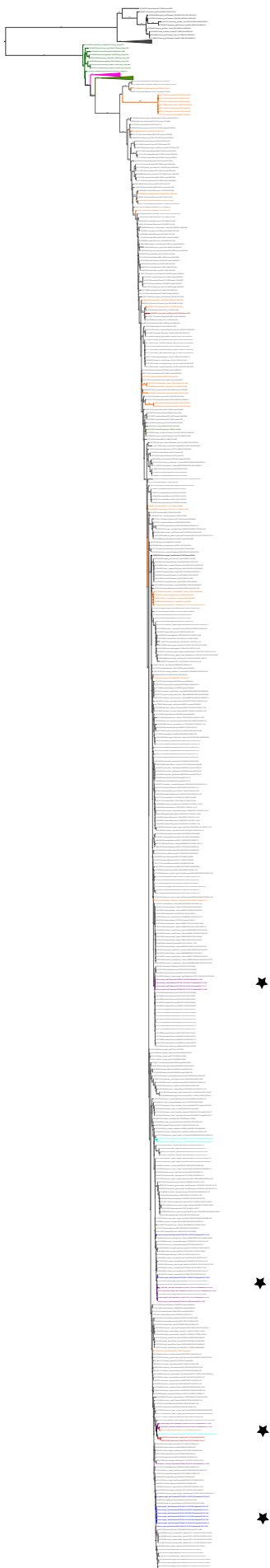

Supplement: FIG S7 [file sph002172264sf7.pdf]
